# Supplementary material for: Plasma Gelsolin Reinforces the Diagnostic Value of FGF-21 and GDF-15 for Mitochondrial Disorders
Source: Int J Mol Sci. 2021 Jun 15;22(12):6396. doi: 10.3390/ijms22126396 (PMC8232645; doi:10.3390/ijms22126396)
Supplement: Supplementary file 1 [file ijms-22-06396-s001.zip › ijms-1253952-supplementary.pdf]

Supplementary Material

Table S1. Main genetic and clinical features of adult patients with mitochondrial disease (MD).

Patient	Sex	Age	OXPHOS enzyme deficiency	Nuclear Gene / mtDNA Variation	Clinical Phenotype	pGSN (µg/mL)	FGF-21 (pg/mL)	GDF-15 (pg/mL)
P1	M	48	Complex I	*OPA1* c.1189A>G	Optic neuropathy and peripheral neuropathy	121.8	226.9	570.7
P2	F	62	Normal	*POLG* [c.752C>T+c.1760C>T] and c.2542G>A	CPEO	373.2	154.7	997.5
P3	M	35	Complex III in the lower normal limit	*POLG* c.2573C>T	Exercise intolerance	125.7	98.9	1098.0
P4	F	45	Normal	*POLG* c.2573C>T	Exercise intolerance	63.0	703.4	1394.0
P5	F	45	Normal	*POLG* c.2864A>G	Exercise intolerance and ptosis	69.5	513.2	1354.0
P6	M	53	Complex I	*POLG* c.2573C>T	Exercise intolerance	87.5	1177.0	1709.0
P7	M	65	High CS	*POLG* [c.752C>T+c.1760C>T] and c.911T>G	SANDO	194.6	524.9	841.6.0
P8	F	71	Normal	*POLG* [c.752C>T+c.1760C>T] and c.2537C>T	SANDO	162.0	2908.0	1865.0
P9	F	19	ND	*RRM2B* c.343G>T and c.817G>A	CPEO, optic neuropathy, ataxia, polyneuropathy	102.6	116.1	2625.0
P10	M	26	ND	*TK2* c.547C>G and c.604_606AAGdel	Myopathy	113.6	486.3	637.8
P11	F	29	Complexes I, III and IV	*TK2* c.323C>T in homozygosis	Myopathy	74.2	205.9	2085.0
P12	F	31	High CS	*TK2* c.323C>T in homozygosis	Myopathy	81.9	138.2	2262.0
P13	F	36	ND	*TK2* c.323C>T in homozygosis	Myopathy	301.5	253.2	2013.0
P14	M	58	Normal	*TK2* c.604_606AAGdel in homozygosis	Myopathy	128.6	743.8	747.4
P15	F	59	Complex III	*TK2* c.604_606AAGdel in homozygosis	Myopathy	100.8	561.4	2978.0
P16	F	60	Normal	*TK2* c.323C>T in homozygosis	Myopathy	960.7	328.8	ND
P17	F	61	ND	*TK2* c.604_606AAGdel in homozygosis	Myopathy	289.6	300.7	2088.0
P18	F	66	Low CS	*TK2* c.604_606AAGdel in homozygosis	Myopathy	673.4	209.1	1297.6
P19	F	76	ND	*TK2* c.604_606AAGdel in homozygosis	Myopathy	390.1	749.7	2488.4
P20	M	58	ND	*TWNK* c.1361T>G	CPEO	173.6	554.7	3491.0

P21	F	67	Normal	*TWNK* c.1070G>C	CPEO	129.9	356.9	518.3
P22	F	79	Complex IV	*TWNK* c.1070G>C	CPEO	108.6	718.4	2095.6
P23	F	18	Complexes I, III, IV	*MTO1* c.1392C>T	Encephalopathy, lactic acidosis and hypertrophic MCP	34.0	503.6	379.7
P24	M	37	ND	*MT-ATP6* m.8993T>G	Retinitis pigmentosa	ND	55.3	472.3
P25	F	70	ND	*MT-ATP6* m.8993T>G	Retinitis pigmentosa and ataxia	138.6	314.7	1332.0
P26	F	30	Normal	*MT-CO1* m.5992G>A (44% heteroplasmy)	Exercise intolerance	102.1	590.9	465.0
P27	F	42	Complex I	*MT-ND5* m.13045A>G (85% heteroplasmy)	MELAS	131.3	81.51	800.9
P28	M	56	Complexes I, III and IV	*MT-TK* m.8344A>G (64% heteroplasmy)	Myopathy	320.0	349.3	2946.0
P29	M	55	ND	*MT-TL1* m.3243A>G	CPEO and cardiopathy	265.8	138.2	1227.0
P30	F	36	Complexes I, III and IV	*MT-TL1* m.3258T>C (96% heteroplasmy)	MELAS	65.2	770.6	1497.0
P31	F	39	ND	*MT-TL1* m.3243A>G (90% heteroplasmy)	MELAS	56.9	212.0	2076.0
P32	M	48	ND	*MT-TL1* m.3243A>G (95% heteroplasmy)	Myopathy	106.9	1168.0	ND
P33	F	76	ND	*MT-TL1* m.3243A>G	Myopathy	65.0	471.9	3883.0
P34	F	41	ND	*MT-TL1* m.3243A>G	Migraines and hearing loss	81.04	328.9	738.2
P35	F	45	ND	*MT-TL1* m.3243A>G (90% heteroplasmy)	Migraines and hearing loss	535.6	192.0	1083.6
P36	F	42	ND	*MT-TL1* m.3243A>G (82% heteroplasmy)	DM and hearing loss	394.4	541.6	3478.0
P37	M	43	Normal	*MT-TL1* m.3243A>G	DM, hearing loss and nephropathy	406.1	460.4	837.9
P38	F	42	ND	*MT-TL1* m.3243A>G	DM and hearing loss	143.0	504.0	3960.4
P39	F	51	ND	*MT-TL1* m.3243A>G (75% heteroplasmy)	DM and hearing loss	130.4	761.5	2546.8
P40	F	---	ND	*MT-TL1* m.A3243>G	DM and hearing loss	85.1	259.7	784.4
P41	F	21	ND	*MT-TL1* m.3243A>G	Exercise intolerance and hearing loss	586.4	518.4	678.1
P42	M	43	Normal	*MT-TL1* m.3243A>G	Ataxia	497.7	746.0	1858.0
P43	F	49	Normal	*MT-TL1* m.3243A>G	CPEO	128.5	567.6	368.4
P44	M	55	ND	*MT-TL1* m.3243A>G	Asymptomatic	353.9	150.3	329.3
P45	F	75	ND	*MT-TL1* m.3243A>G	DM	369.9	71.2	1804.0
P46	M	44	ND	*MT-TL1* m.3243A>G	Hearing loss	532.7	120.0	654.8

P47	M	45	Normal	*MT-TN* m.5688 T>C (70% heteroplasmy)	Myopathy	209.7	420.0	2568.0
P48	M	41	ND	*MT-TN* m.5692T>C (80% heteroplasmy)	Exercise intolerance	63.5	532.5	2643.0
P49	M	18	Normal	Single mtDNA deletion (4.8 kb, 60% heteroplasmy)	CPEO	69.5	272.5	794.8
P50	M	30	Complex IV	Single mtDNA deletion (4.9 Kb, 59% heteroplasmy)	CPEO	245.2	126.4	1327.0
P51	F	38	Normal	Single mtDNA deletion (5.7 kb, 34% heteroplasmy)	CPEO and exercise intolerance	522.4	389.0	1777.0
P52	F	38	Complexes I, III, IV	Single mtDNA deletion (3.4 kb, 68% heteroplasmy)	CPEO and myopathy	215.8	230.4	5047.0
P53	M	52	Normal	Single mtDNA deletion (4 kb, 78% heteroplasmy)	CPEO	196.8	318.7	1875.0
P54	F	---	High CS	Single mtDNA deletion (4.7 kb, 40% heteroplasmy)	CPEO and myopathy	335.7	457.0	1504.0
P55	F	20	Complex I	Single mtDNA deletion (4.9 kb, 75% heteroplasmy)	KSS	155.0	124.2	4128.0
P56	F	24	Normal	Single mtDNA deletion (7.1 kb, 60% heteroplasmy)	KSS	106.7	119.7	2093.0
P57	M	63	Normal	Multiple mtDNA deletions	CPEO	693.6	307.1	1943.6
P58	F	40	Complexes I, IV in the lower normal limit	Multiple mtDNA deletions	Focal myopathy	154.0	711.7	2292.0
P59	F	57	Normal	Multiple mtDNA deletions	Myopathy	141.4	86.8	2229.0
P60	M	41	Normal	Multiple mtDNA deletions	Myopathy	284.9	624.3	2344.0

pGSN: plasma GSN; M: Male; F: Female; ND: Not determined; CS: Citrate synthase; CPEO: Chronic Progressive External Ophthalmoplegia; SANDO: Sensory Ataxic Neuropathy, Dysarthria, and Ophthalmoparesis; MELAS: Mitochondrial Encephalopathy, Lactic Acidosis, and Stroke-like episodes; DM: Diabetes Mellitus; MCP: Miocardiomyopathy; KSS: Kearns-Sayre syndrome. *OPA1* (NM_130837), *POLG* (NM_001126131), *RRM2B* (NM_015713), *TK2* (NM_004614); *TWNK* (NM_021830), *MTO1* (NM_012123.3). % heteroplasmy in muscle, when available.

Table S2. Main genetic and clinical features of adult patients with non-mitochondrial disease (non-MD).

Patient	Sex	Age	Clinical Phenotype	pGSN (μg/mL)	FGF-21 (pg/mL)	GDF-15 (pg/mL)
NP1	M	58	Amyotrophic lateral sclerosis	143.0	171.1	3452.0
NP2	M	58	Amyotrophic lateral sclerosis	170.5	485.6	1705.0
NP3	F	60	Amyotrophic lateral sclerosis	288.0	49.9	877.1
NP4	F	75	Amyotrophic lateral sclerosis	65.9	166.8	1804.0
NP5	F	79	Amyotrophic lateral sclerosis	106.7	99.5	1139.0
NP6	F	18	CPEO	97.1	2.0	298.0
NP7	M	64	CPEO plus	240.0	100.5	1037.0
NP8	M	16	Exercise intolerance	120.4	0.6	387.9
NP9	F	18	Exercise intolerance	360.3	106.8	361.9
NP10	M	28	Exercise intolerance	107.2	927.2	322.6
NP11	F	41	Exercise intolerance	391.6	121.8	295.2
NP12	F	55	Exercise intolerance	950.3	34.2	354.8
NP13	F	58	Exercise intolerance	109.8	2.01	689.5
NP14	F	61	Exercise intolerance	69.7	131.7	466.6
NP15	M	63	Exercise intolerance (McArdle disease)	137.1	6.1	492.5
NP16	M	40	HyperCKemia	541.2	141.5	245.8
NP17	F	48	HyperCKemia (*ANO5* mutation)	172.2	91.8	477.1
NP18	M	50	HyperCKemia	125.1	79.6	659.5
NP19	M	50	HyperCKemia	153.6	9.3	634.6
NP20	F	53	HyperCKemia (dominant *CAPN3* mutation)	395.2	675.9	5880.0
NP21	M	54	HyperCKemia (SAHS)	84.2	504.2	536.9
NP22	M	56	HyperCKemia (*ANO5* mutation)	112.2	444.4	484.9
NP23	F	67	HyperCKemia	198.5	177.6	957.3
NP24	F	70	Myasthenia	455.6	21.4	1430.0
NP25	M	78	Myasthenia	450.2	51.7	1254.0
NP26	F	24	Myopathy (immune-mediated)	364.5	702.7	977.3
NP27	M	30	Myopathy (Pompe disease)	129.4	24.3	396.8
NP28	M	36	Myopathy (oculopharyngeal dystrophy)	375.5	622.9	397.6

NP29	F	49	Myopathy (toxic)	319.7	6.6	6704.0
NP30	F	63	Myopathy (*DYSF* muscle disease)	343.9	46.4	800.5
NP31	M	67	Myopathy	363.2	219.0	1061.6
NP32	F	75	Myopathy	63.4	1.0	1668.0
NP33	F	--	Myopathy	1223.0	1867.0	1026.4
NP34	M	21	Rhabdomyolysis	374.9	82.2	449.5
NP35	M	27	Rhabdomyolysis	391.2	35.7	304.6
NP36	F	28	Rhabdomyolysis. CKD.	250.3	130.6	5956.0
NP37	M	29	Rhabdomyolysis	799.8	39.3	342.2
NP38	F	35	Rhabdomyolysis	128.6	21.4	307.8
NP39	M	36	Rhabdomyolysis	58.8	129.5	357.5
NP40	F	40	Rhabdomyolysis	852.1	420.0	550.6
NP41	M	44	Rhabdomyolysis	329.3	236.1	303.1

pGSN: plasma GSN; M: Male; F: Female; CPEO: Chronic Progressive External Ophthalmoplegia; SAHS (apnea-hypopnea syndrome); CKD: chronic kidney disease.

Table S3. Sex, age and biomarkers plasma levels in the healthy controls presented in this work.

Control	Sex	Age	pGSN (µg/mL)	FGF-21 (pg/mL)	GDF-15 (pg/mL)
C1	M	21	459.9	58.9	844.8
C2	M	24	329.8	206.7	630.8
C3	M	25	446.8	139.2	265.3
C4	M	46	497.7	222.0	225.0
C5	M	49	614.1	95.8	341.9
C6	M	50	474.1	259.9	747.5
C7	M	50	602.8	178.4	552.4
C8	M	52	645.4	74.3	417.3
C9	M	53	213.4	299.7	376.3
C10	M	54	500.9	320.9	525.4
C11	M	55	534.1	101.8	520.7
C12	M	56	579.6	194.5	593.1
C13	M	57	620.0	264.0	583.6
C14	M	58	376.2	242.6	432.0
C15	M	59	393.2	62.5	547.6
C16	M	59	537.3	116.8	577.1
C17	M	64	483.9	35.2	827.7
C18	M	65	297.2	178.4	548.4
C19	M	66	326.3	458.2	505.0
C20	M	66	829.6	54.4	545.2
C21	M	68	529.0	64.3	459.5
C22	M	68	348.1	282.0	570.7
C23	M	73	336.9	384.1	1563.0
C24	M	74	379.8	164.8	901.2
C25	M	75	272.1	104.5	1423.0
C26	F	18	330.8	278.4	489.2
C27	F	18	301.6	26.1	152.8
C28	F	19	277.0	43.4	234.0

C29	F	20	414.6	59.1	371.0
C30	F	20	267.0	134.6	450.8
C31	F	22	432.1	100.4	327.6
C32	F	23	343.2	168.3	563.8
C33	F	23	392.9	156.0	312.8
C34	F	23	313.7	152.8	509.6
C35	F	24	259.1	91.0	230.9
C36	F	24	441.3	191.1	287.3
C37	F	25	357.3	137.6	184.2
C38	F	25	412.4	55.0	536.0
C39	F	47	360.7	111.2	363.1
C40	F	47	339.2	100.9	474.2
C41	F	51	376.2	338.8	594.7
C42	F	53	363.2	107.9	314.8
C43	F	55	523.3	143.1	377.1
C44	F	57	607.0	456.8	774.7
C45	F	57	444.2	242.7	1186.0
C46	F	61	334.5	186.2	610.0
C47	F	64	261.9	383.6	947.9
C48	F	66	507.3	250.0	953.0
C49	F	69	438.2	24.5	662.1
C50	F	70	404.9	331.3	786.6
C51	F	70	364.3	727.0	810.5
C52	F	71	529.4	150.7	1116.0
C53	F	73	813.6	228.0	818.0
C54	F	74	374.7	151.4	684.4
C55	F	74	633.4	39.4	841.4
C56	F	75	356.2	359.3	418.5

pGSN: plasma GSN; M: Male; F: Female.
